# Intercrops Maintain Orchard Soil Nutrients Accumulation with Variation in Soil Microbiome Composition and Function

**DOI:** 10.3390/plants15132030

**Published:** 2026-06-30

**Authors:** Congyi Zhu, Yongjing Huang, Chaochen Tang, Mingyang Sun, Yang Hu, Xiuting Xu, Jingzhao Liu, Pingzhi Wu, Ruimin Zhang, Jiwu Zeng

**Affiliations:** 1 Institute of Fruit Tree Research, Guangdong Academy of Agricultural Sciences, Key Laboratory of South Subtropical Fruit Biology and Genetic Resource Utilization (MOA), Guangdong Province Key Laboratory of Science and Technology Research on Fruit Tree, Guangzhou 510640, China; zhucongyi@gdaas.cn (C.Z.);; 2 Institute of Crops Research, Guangdong Academy of Agricultural Sciences, Guangdong Province Key Laboratory of Crop Genetic Improvement, Guangzhou 510640, China; 3 Xinhui Forestry Science Institute, Jiangmen 529100, China; huyangchong@126.com (Y.H.); 13630452546@163.com (X.X.); 13612299104@163.com (J.L.)

**Keywords:** intercropping, soybean, sweet potato, microbiome, citrus

## Abstract

The intercropping system is used for weed control in orchards, but the intercrops need to be well-designed to fit into the row spaces of fruit trees. In this study, the citrus (*Citrus reticulata* cv. *Chachiensis*) row spaces were intercropped with either soybean (*Glycine max* (L.) Merr.) or sweet potato (*Ipomoea batatas* (L.) Lam.), and their effects on weed control, soil physiochemical properties, and soil microbiome were compared to the natural weeds. Both plant species were effective in reducing the orchard weeds, and their different varieties commonly improved soil organic matter, available P and K, and beneficial metal elements compared to the weeds. Even though the soil fungal and bacterial richness and diversity of the intercrops were not significantly altered, their composition, structure, and function were distinctive to those of the weeds. The soils of the intercrops generally enriched with the fungal genera of *Talaromyces* and *Penicillium* and the bacterial genera *Sphingomonas*, *Knoellia*, and *Nocardioides*. Accordingly, the altered microbial communities, in taxonomy, correlated to the enriched cellular functional pathways of glycolysis and gluconeogenesis, homologous recombination, nitrogen metabolism, lipoic acid metabolism, mismatch repair, DNA replication, nicotinate and nicotinamide metabolism. Taken together, these results imply that intercrops and weeds exert distinct effects on soil nutrient accumulation, and these effects are associated with their differential impacts on soil microbiomes—which are likely driven by the rhizosphere activities of the intercrops.

## 1. Introduction

Intercropping, often synonymous with mixed cropping or polyculture, is a traditional farming practice [1,2]. According to the spatial and temporal intensification of different crops, intercropping can be classified into different types of systems, including row intercropping, mixed intercropping, strip intercropping, and relay intercropping [2,3]. Of which, row intercropping is a common practice and easy to operate; the base crops are grown in regular rows and the intercrops are sown within the row space [4,5]. In intercropping systems, the concurrently growth of two or more crops can improve the utilization efficiency of agricultural inputs, such as intensification of farmland crops, reduction of waste fertilizers, ecological control of farmland weeds, and enhancement of crop yield [1,4,5]. Farmland weeds are a serious threat to crop production, they interfere with crop growth through the competition for soil nutrients, transmission of plant pests and diseases, and alteration soil beneficial microorganisms [6,7]. To achieve a good performance, an intercropping system should be well designed, and appropriate crop selection is needed to avoid interspecific competition both among the crops and with adjacent weeds [1,8].

In previous agricultural practices, leguminous crops have been proved as great component crops in intercropping systems [9,10]. Leguminous crops, with their symbiosis between *Rhizobium* bacteria, can fix atmospheric nitrogen biologically [9]. Legumes usually demand less nitrogen compared to other crops like corns and cereals, their fixed nitrogen is not only used for their own nutrition, but also for the associated non-leguminous crops [11,12]. For instance, the legumes biologically fixed nitrogen of 80 to 350 kg ha^−1^ year^−1^, and up to 15% of the fixed nitrogen was shared to cereals, thus largely reducing the fertilizer input in the intercropping system of cereal and legume [13,14]. Except for nitrogen, the availability of soil organic carbon and other soil elements like phosphorus, potassium, and sulfur also increased in the legume-based intercropping systems due to more diverse root exudates, improved soil nitrogen availability, and altered soil microbial population and activities [15,16]. Recently, a two-year study of soybean intercropping in citrus orchards revealed multifaceted effects, including enhanced citrus fruit quality, increased soil nutrient accumulation, and reshaped soil bacterial communities [17]. In our practice, however, the low planting density required for soybean allowed weed persistence, thereby undermining its efficiency as an intercrop. Therefore, more suitable intercrops still need to be selected based on the specific characteristics of different plant species and their growth environments.

Sweet potato (*Ipomoea batatas* (L.) Lam.) is an ideal intercrop, due to its high yield potential and low input requirement. For example, it is widely intercropped with maize (*Zea mays* L.) in the mountainous regions of Southwestern China, contributing approximately 450,000 hectares, or about half, to the total sweet potato planting area in Chongqing Municipality and Sichuan Province [18]. In addition, the intercropping with sweet potato is also beneficial in improving soil ecosystem function, such as improving soil aggregates, soil microbial biomass, and microbial enzyme activities [19]. To date, however, the effectiveness of sweet potato as an intercrop with citrus remains largely untested. In this study, we established row intercropping systems in an orchard of perennial citrus (*Citrus reticulata* cv. *Chachiensis*) by introducing either a leguminous plant species, soybean (*Glycine max* (L.) Merr.), or a non-leguminous plant species, sweet potato (*Ipomoea batatas*), into the row spaces. Each plant species was planted with two representative varieties. We tested the hypothesis that intercropping citrus with soybean or sweet potato improves soil nutrient availability and simultaneously drives convergent shifts in the taxonomic and functional structures of both soil fungal and bacterial communities.

## 2. Materials and Methods

### 2.1. Planting of Intercrops

**Figure 1:**
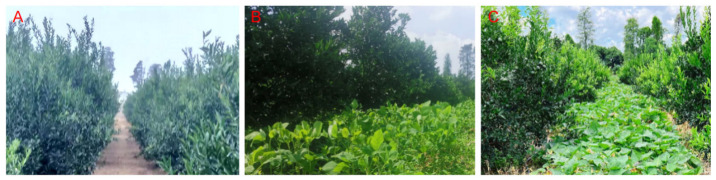
Citrus row space cleared (**A**), and intercropped with soybeans (**B**) or sweet potatoes (**C**).

The field experiment was carried out in a citrus orchard in Bei’an (22.43° N, 113.03° E), Jiangmen City, Guangdong Province. The base crop was an edible and medicinal citrus (*Citrus reticulata* cv. *Chachiensis*), which is an important horticultural crop in Guangdong [20,21]. The soil in the orchard is a gleyed paddy soil with acidic pH and a texture ranging between sandy loam and loam. The citrus plants were grown in rows, the row spacing was five meters wide, and the plant spacing was 2.5 m. In the early March of 2023, a total of 26 rows of citrus, including 25 row spaces (space between the rows, about 25 m long × 5 m wide), were selected for our intercropping experiment. The soils of the row spaces were tilled, and all the wild plants were removed (Figure 1A). The cuttings of two sweet potato varieties, Guangshu87 (GS87) and Pushu32 (PS32), and the seedlings of two soybean varieties, Huning95-1 (HN95-1) and Hongqiu1 (HQ1), were transplanted from a greenhouse at Bei’an and subsequently planted in five-row plots per variety, with a randomized arrangement. The other five control row spaces did not grow any intercrops. For the sweet potatoes, only one row was planted in the middle of each row space, their plant spacing was 30 cm. For the soybeans, three rows were planted in each row space, their plant spacing was 10 cm. No pesticides, herbicides, or biofertilizers were applied to the citrus, intercrops, or weeds. In May, at the young fruit stage, all citrus trees received a uniform foliar application of GaiMeiDuo (Sichuan Shifang Demei Industrial Co., Ltd., Shifang, Sichuan, China), PengErMei (OMEX Agrifluids, King’s Lynn, UK), and potassium dihydrogen phosphate (Hebei Fangzhou Agricultural Technology Co., Ltd., Shijiazhuang, Hebei, China).

### 2.2. Plant Investigation and Soil Sampling

On 11 July 2023, three 1 × 1 m^2^ plots were randomly drawn in each row spacing, the numbers of all plant species in the plots were recorded. The soils sampled from the three plots in a row spacing were mixed thoroughly to form a sample. In each plot, plant debris was removed from the soil surface, the topsoil from 0 to 10 cm depth was collected using a stainless-steel spade. Soils were put in a plastic bag, and moved back to the laboratory as soon as possible.

### 2.3. Soil Physicochemical Analyses

Soil pH was determined using a pH meter (Mettler-Toledo FE20, Greifensee, Switzerland). Next, soils were air-dried at room temperature (25 °C) and passed through a 1-mm sieve for physicochemical analyses. Soil organic matter was measured using a Vario MACRO cube organic elemental analyzer (Elementar, Langenselbold, Germany). Soil N, P, K, and other elements were determined by wet digestion and inductively coupled plasma atomic emission spectroscopy, namely ICP-AES, in Hangzhou, China [22,23].

### 2.4. Amplicon Sequencing

The total DNA of soil fungi and bacteria was extracted from 250 mg soils using the E.Z.N.A.^®^ soil DNA Kit (Omega Bio-tek, Norcross, GA, USA) according to manufacturer’s instructions. The fungal and bacterial barcoding sequences of the fungal nuclear ribosomal internal transcribed spacer (ITS rDNA) and bacterial 16S rRNA were amplified with the primer sets ITS1F (5′-CTTGGTCATTTAGAGGAAGTAA-3′)/ITS2R (5′-TGTGTTCTTCATCGATG-3′) and 338F (5′-ACTCCTACGGGAGGCAGCAG-3′)/806R (5′-GGACTACHVGGGTWTCTAAT-3′), respectively [24,25]. The 20 μL volume of PCR reaction mixture was as follows: 4 μL 5 × Fast Pfu buffer, 2 μL 2.5 mM dNTPs, 0.4 μL each primer (10 μM), 0.4 μL Fast Pfu polymerase, and 10 ng of template DNA. The PCR thermal program for fungal ITS was as follows: initial 94 °C for 5 min, followed by 30 cycles of 94 °C for 30 s, 52 °C for 30 s, 72 °C for 30 s, and a final extension at 72 °C for 10 min; for bacterial 16S rRNA the program was as follows: initial 95 °C for 3 min, followed by 27 cycles of 95 °C for 30 s, 55 °C for 30 s, 72 °C for 45 s, and a final extension at 72 °C for 10 min. The PCR product was verified on 2% agarose gel and purified using the PCR Clean-Up Kit (Yuhua, Shanghai, China). The purified amplicons were quantified using Qubit 4.0 (Thermo Fisher Scientific, Waltham, MA, USA), and then, pooled in equimolar amounts for paired-end sequencing on the Illumina Nextseq2000 platform (Illumina, San Diego, CA, USA).

The raw sequencing reads were filtered using fastq version 0.19.6 by deleting reads that (i) had an average quality score of <20 over a 50 bp sliding window, (ii) overlapping paired-end sequences < 10 bp, or (iii) >2 nucleotide mismatches in primer matching [26,27]. The paired-end sequences were merged using FLASH v. 1.2.7 (http://ccb.jhu.edu/software/FLASH/ (accessed on 20 March 2024)) and clustered into operational taxonomic units (OTUs) at 97% sequence similarity level using UPARSE v. 7.1 [28]. The fungal and bacterial OTUs were annotated using the RDP Classifier v. 2.2 against the UNITE (https://unite.ut.ee/ (accessed on 21 March 2024)) and Silva v. 138 (https://www.arb-silva.de/ (accessed on 21 March 2024)) databases, respectively.

### 2.5. Metagenomic Sequencing

Microbial DNA was fragmented to about 400 bp using Covaris M220 (Gene Company Limited, Hong Kong SAR, China) and used to construct the sequencing library using NEXTFLEX Rapid DNA-Seq (Bioo Scientific, Austin, TX, USA). The sequencing was performed on Illumina NovaSeq platform (Illumina, San Diego, CA, USA) at Majorbio Bio-Pharm Technology Co., Ltd. (Shanghai, China). The low quality reads, with length < 50 bp or with a quality value < 20 or having N bases, were removed by fastp 0.20.0 [29] and then assembled using MEGAHIT v. 1.1.2 (https://github.com/voutcn/megahit (accessed on 10 April 2024)). Contigs with a final length ≥300 bp were kept and used for gene prediction and annotation. Non-redundant sequences were annotated by submitting to the Kyoto Encyclopedia of Genes and Genomes database (v. 0.8.35, https://www.genome.jp/kegg/ (accessed on 10 April 2024)).

### 2.6. Statistical Analysis

The OTU table of amplicon sequencing was generated in QIIME2 2020.6 [30]. The number of sequences of different samples was then normalized to the least sequence number, and subjected to the calculation of alpha diversity indices (Observed species, Shannon, Simpson, ACE, Chao1, Good’s coverage, and Phylogenetic diversity). The further statistical analyses were conducted using the R software v. 4.0.0 (http://www.r-project.org/ (accessed on 27 March 2024)). Differences in the microbial community structure among different intercropping treatments were assessed by performing PERMANOVA with 999 permutations in the vegan package [31]. A principal components analysis (PCoA) based on Bray–Curtis dissimilarities was performed with the vegan package, and the results were plotted using ggplot2 [32,33]. The correlation analysis was performed with the psych package v. 2.6.1 (https://personality-project.org/r/psych/ (accessed on 27 March 2024)) and was plotted with the ggcorrplot2 package v. 0.1.4.1 (https://github.com/caijun/ggcorrplot2 (accessed on 27 March 2024)). ANOVA, followed by Tukey post-hoc pairwise test, was used for comparisons among the intercropping treatments.

## 3. Results

### 3.1. Effects of Intercropping on Soil Physicochemical Properties and Metal Contents

In the blocks of weeds, some common weeds in Guangdong, such as *Bidens pilosa* L. (2.41 ± 0.15 plants per m^2^), *Oxalis corniculata* L. (10.66 ± 2.5), *Eleusine indica* (L.) Gaertn. (8.14 ± 3.28), *Alternanthera sessilis* (L.) DC. (3.56 ± 1.77), *Digitaria sanguinalis* (L.) Scop. (8.92 ± 5.66), *Solanum nigrum* L. (2.11 ± 0.05), and *Setaria viridis* (L.) Beauv. (12.56 ± 5.89), were found as the most dominant weeds. In the blocks of intercrops, soybeans and sweet potatoes dominated in their own blocks, respectively (Figure 1B,C). From the results of soil chemical properties, the soils of intercrops consistently showed significantly higher contents of P, Mg, Mn, and Zn than the soils of weeds (*p* < 0.05, Table 1). In addition, the soils of intercrops commonly showed significantly higher contents of organic matter (except GS87), K (except PS32), and Ca (except HN95-1) than the soils of weeds (*p* < 0.05, Table 1). On average, the soils of intercrops also showed higher contents of N, Fe, Na, and Cu than the soils of weeds (Table 1).

### 3.2. Effects of Intercropping on Soil Microbial Richness, Diversity and Composition

**Figure 2:**
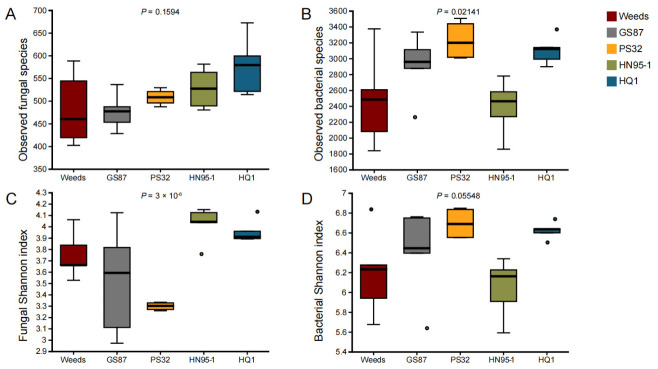
Comparison of the fungal and bacterial alpha diversity in the soils of intercrops and weeds. (**A**,**B**), the number of observed fungal (**A**) and bacterial (**B**) species. (**C**,**D**) The Shannon diversity index of fungal (**C**) and bacterial (**D**) communities.

The observed fungal (Figure 2A) and bacterial species (Figure 2B), and also their Shannon index in soils (Figure 2C and Figure 2D, respectively), were not significantly different between any of the intercrops and the weeds. Within intercrops, the observed bacterial species value was significantly different (*p* < 0.05, Figure 2B) between PS32 (3226 ± 249.5) and HN95-1 (2389 ± 350.6), the fungal Shannon index was significantly different (Figure 2C) between GS87 (3.5 ± 0.5) and HN95-1 (4 ± 0.2, *p* < 0.05), PS32 (3.3) and HN95-1 (*p* < 0.01), and PS32 and HQ1 (4 ± 0.1, *p* < 0.01).

**Figure 3:**
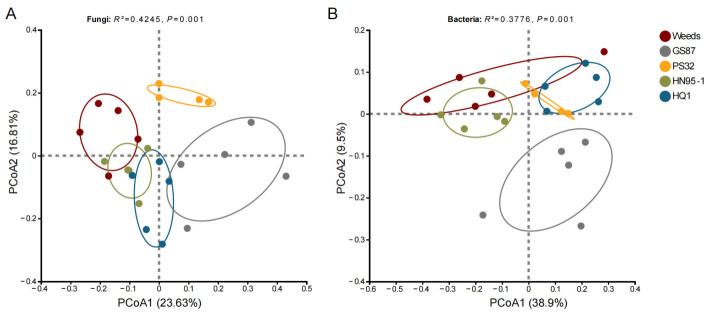
Comparison of the fungal and bacterial communities in the soils of intercrops and weeds. (**A**,**B**), plot of the first two principal co-ordinates after principal co-ordinate analysis (PCoA) based on the Bray–Curtis distances of fungal (**A**) and bacterial (**B**) communities, respectively.

Based on the principal co-ordinates analysis (PCoA), the soil fungal communities of intercrops deviated significantly from the weeds mainly along PCoA 1 (23.6%, Figure 3A), and the soil bacterial communities of intercrops deviated significantly from the weeds mainly along PCoA 2 (9.5%, Figure 3B). Notably, both the soil fungal and bacterial communities of the intercrop GS87 were most distinctive from other samples, implying its unique composition and structure of microbial communities.

### 3.3. Effects of Intercropping on Soil Microbial Top Taxa

**Figure 4:**
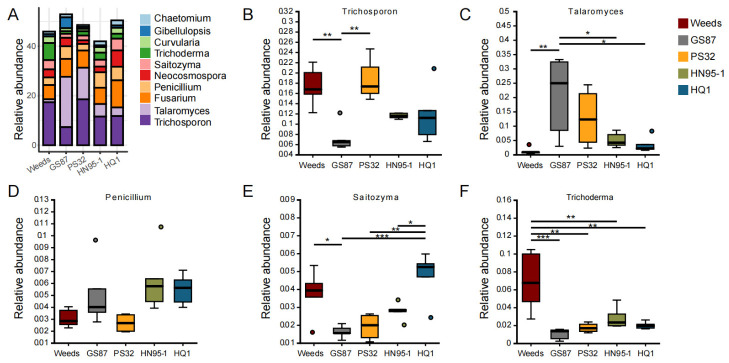
Comparison of the relative abundances of the top 10 fungal genera in the soils of intercrops and weeds. (**A**) Stack plot of all the top 10 genera; (**B**) *Trichosporon*; (**C**) *Talaromyces*; (**D**) *Penicillium*; (**E**) *Saitozyma*; and (**F**) *Trichoderma*. The significant *p* values of paired comparisons are marked above each box: “*” for *p* < 0.05, “**” for *p* < 0.01, and “***” for *p* < 0.001.

The top 10 fungal genera of soil samples were *Trichosporon*, *Talaromyces*, *Fusarium*, *Penicillium*, *Neocosmospora*, *Saitozyma*, *Trichoderma*, *Curvularia*, *Gibellulopsis*, and *Chaetomium* (Figure 4A), which consisted of 42 to 52.9% of the relative abundance (the proportion of sequences) in the soil fungal communities. In comparison to the weeds, the relative abundance of the genera *Trichosporon* (except PS32, Figure 4B), *Saitozyma* (except HQ1, Figure 4E), and *Trichoderma* (Figure 4F) generally decreased, and of the genera *Talaromyces* (Figure 4C) and *Penicillium* (except PS32, Figure 4D) generally increased, in soil fungal communities of the intercrops. Especially, these genera were highly affected by the intercrop GS87. The relative abundance of *Trichosporon* decreased from 17.4 ± 3.8 in the weeds to 7.3 ± 2.8 in GS87 (*p* < 0.01), of *Saitozyma* decreased from 3.7 ± 1.4 to 1.6 ± 0.3 (*p* < 0.05), and of *Trichoderma* decreased from 6.9 ± 3.3 to 1 ± 0.6 (*p* < 0.001), while of *Talaromyces* increased from 1.2 ± 1.3 to 20.4 ± 13.9 (*p* < 0.01).

**Figure 5:**
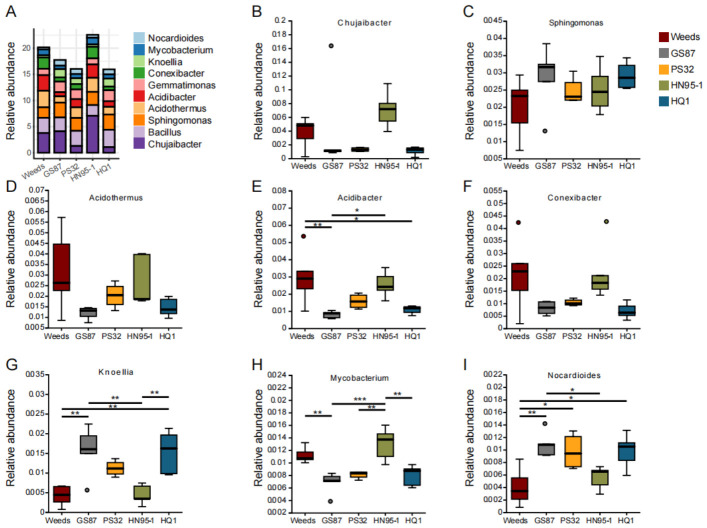
Comparison of the relative abundances of the top 10 bacterial genera in the soils of intercrops and weeds. (**A**) Stack plot of all the top 10 genera; (**B**) *Chujaibacter*; (**C**) *Sphingomonas*; (**D**) *Acidothermus*; (**E**) *Acidibacter*; (**F**) *Conexibacter*; (**G**) *Knoellia*; (**H**) *Mycobacterium*; and (**I**) *Nocardioides*. The significant *p* values of paired comparisons are marked above each box: “*” for *p* < 0.05, “**” for *p* < 0.01, and “***” for *p* < 0.001.

Similarly, the top 10 bacterial genera of soil samples were *Chujaibacter*, *Bacillus*, *Sphingomonas*, *Acidothermus*, *Acidibacter*, *Gemmatimonas*, *Conexibacter*, *Knoellia*, *Mycobacterium*, and *Nocardioides* (Figure 5A), which consisted of 16 to 22.6% of the relative abundance in the soil bacterial communities. In comparison to the weeds, the relative abundance of the genera *Chujaibacter* (except HN95-1, Figure 5B), *Acidothermus* (Figure 5D), *Acidibacter* (Figure 5E), *Conexibacter* (Figure 5F), and *Mycobacterium* (except HN95-1, Figure 5H) generally decreased, and of the genera *Sphingomonas* (Figure 5C), *Knoellia* (except HN95-1, Figure 5G), and *Nocardioides* (Figure 5I) generally increased, in soil bacterial communities of the intercrops. Especially, these genera were highly affected by the intercrops GS87 and HQ1. For instance, the relative abundance of *Acidibacter* decreased from 3 ± 1.6 in the weeds to 0.8 ± 0.2 in GS87 (*p* < 0.01), of *Mycobacterium* decreased from 1.1 ± 0.1 to 0.7 ± 0.2 (*p* < 0.01), while of *Knoellia* increased from 0.4 ± 0.3 to 1.6 ± 0.6 (*p* < 0.01), of *Nocardioides* increased from 0.4 ± 0.3 to 1.1 ± 0.2 (*p* < 0.01). The relative abundance of *Acidibacter* decreased from 3 ± 1.6 in the weeds to 1.1 ± 0.2 in HQ1 (*p* < 0.05), while of *Knoellia* increased from 0.4 ± 0.3 to 1.5 ± 0.5 (*p* < 0.01), and of *Nocardioides* increased from 0.4 ± 0.3 to 1 ± 0.3 (*p* < 0.01).

### 3.4. Correlation Between Soil Physicochemical Properties and Microbial Communities

**Figure 6:**
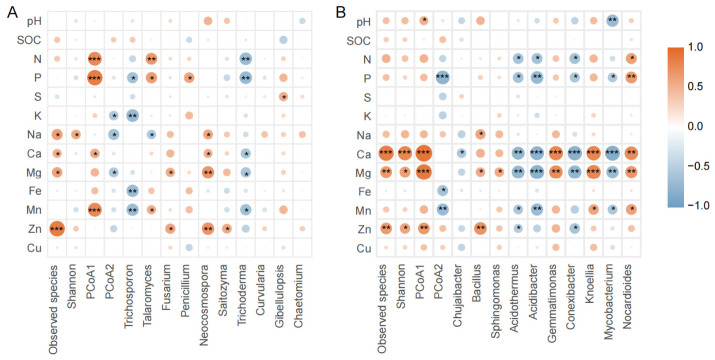
Correlation between soil physicochemical properties, metal contents, and microbial communities. (**A**) Fungal communities and (**B**) bacterial communities. The correlation coefficients are represented by different colors, the significant *p* values are marked above each circle: “*” for *p* < 0.05, “**” for *p* < 0.01, and “***” for *p* < 0.001.

The correlations between soil properties and microbial communities were stronger for bacteria than for fungi (Figure 6A vs. Figure 6B). For example, N and P, as important soil nutrients, positively correlated to three soil fungal genera of *Talaromyces* (*R*^2^ = 0.66, *p* < 0.01 for N; *R*^2^ = 0.64, *p* < 0.05 for P) and *Penicillium* (*R*^2^ = 0.27, not significant; *R*^2^ = 0.6, *p* < 0.05), and one bacterial genus of *Nocardioides* (*R*^2^ = 0.62, *p* < 0.05; *R*^2^ = 0.66, *p* < 0.01). On the contrary, N and P negatively correlated to one fungal genus of *Trichoderma* (*R*^2^ = −0.7, *p* < 0.01; *R*^2^ = −0.71, *p* < 0.01), and four bacterial genera of *Acidothermus* (*R*^2^ = −0.61, *p* < 0.05; *R*^2^ = −0.61, *p* < 0.05), *Acidibacter* (*R*^2^ = −0.62, *p* < 0.05; *R*^2^ = −0.71, *p* < 0.01), *Conexibacter* (*R*^2^ = −0.62, *p* < 0.05; *R*^2^ = −0.58, *p* < 0.05), and *Mycobacterium* (*R*^2^ = −0.34, not significant; *R*^2^ = −0.55, *p* < 0.05). Among all the metal elements, Ca, Mg, and Zn were more related to the microbial communities, especially to the bacterial communities. For example, these elements significantly correlated with bacterial observed species (*R*^2^ = 0.84, *p* < 0.001 for Ca; *R*^2^ = 0.67, *p* < 0.01 for Mg; *R*^2^ = 0.66, *p* < 0.01 for Zn), Shannon index (*R*^2^ = 0.79, *p* < 0.001; *R*^2^ = 0.62, *p* < 0.05; *R*^2^ = 0.6, *p* < 0.05), and PCoA 1 (*R*^2^ = 0.92, *p* < 0.001; *R*^2^ = 0.88, *p* < 0.001; *R*^2^ = 0.66, *p* < 0.01). In addition, Ca and Mg positively correlated to the bacterial genera of *Gemmatimonas* (*R*^2^ = 0.79, *p* < 0.001 for Ca; *R*^2^ = 0.76, *p* < 0.01 for Mg), *Knoellia* (*R*^2^ = 0.8, *p* < 0.001; *R*^2^ = 0.8, *p* < 0.001), and *Nocardioides* (*R*^2^ = 0.76, *p* < 0.01; *R*^2^ = 0.68, *p* < 0.01), and negatively correlated to the bacterial genera of *Acidothermus* (*R*^2^ = −0.72, *p* < 0.01; *R*^2^ = −0.72, *p* < 0.01), *Acidibacter* (*R*^2^ = −0.77, *p* < 0.001; *R*^2^ = −0.79, *p* < 0.001), *Conexibacter* (*R*^2^ = −0.8, *p* < 0.001; *R*^2^ = −0.75, *p* < 0.01), and *Mycobacterium* (*R*^2^ = −0.83, *p* < 0.001; *R*^2^ = −0.7, *p* < 0.01).

### 3.5. Effects of Intercropping on Soil Microbial Community Functions

**Figure 7:**
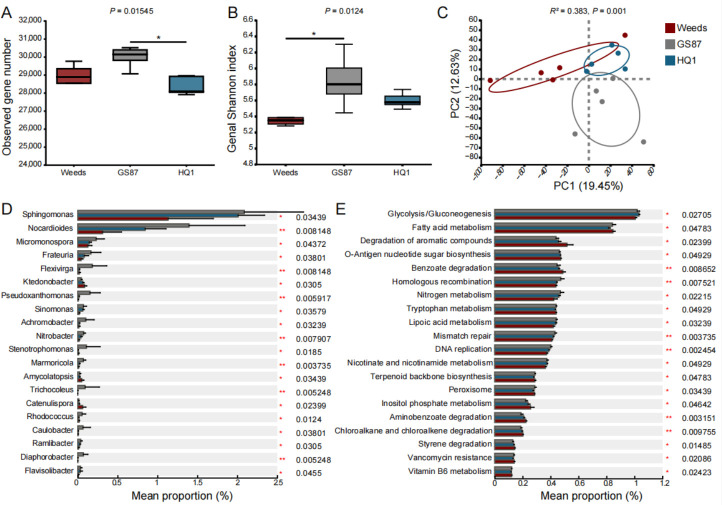
Comparison of the detected genes and their functions in the soils of intercrops and weeds. (**A**) The number of genes; (**B**) the Shannon diversity index of genes; (**C**) plot of the first two principal components after the principal component analysis (PCA) based on the genes; and (**D**,**E**) the bacterial genera (**D**) and cellular functional pathways (**E**) annotated from the detected genes. The significant *p* values of comparisons within (**D**,**E**) are marked at the end of each bar: “*” for *p* < 0.05 and “**” for *p* < 0.01.

The soil samples of GS87, HQ1 and the weeds were subjected to metagenomic sequencing. The number of detected genes was significantly different between GS87 and HQ1 (*p* < 0.05, Figure 7A). The diversity of detected genes, represented by the Shannon index, was higher in the intercrops (GS87: 5.8 ± 0.3, HQ1: 5.6 ± 0.1) than in the weeds (5.4 ± 0.1, versus GS87, *p* < 0.05; Figure 7B). The structure and composition of detected genes was significantly different among the intercrops and weeds (*p* < 0.01, Figure 7C). The samples of GS87, HQ1, and the weeds could be differentiated mainly along the principal component 2 (12.6%).

At the genus level, the bacterial species from over ten genera significantly contributed more genes (the proportion of genes, %) in the soil microbial communities of intercrops than their counterparts of weeds (Figure 7D), such as *Sphingomonas* (GS87: 2.1 ± 0.7, HQ1: 2 ± 0.3, weeds: 1.1 ± 0.6, *p* < 0.05), *Nocardioides* (1.4 ± 0.7, 0.8 ± 0.3, 0.3 ± 0.2, *p* < 0.01), and *Micromonospora* (0.2 ± 0.1, 0.1, 0.1, *p* < 0.05). Accordingly, the enriched genes by these bacterial genera in the soil microbial communities of intercrops correlated to the enriched cellular functional pathways (Figure 7E), such as glycolysis and gluconeogenesis (1.1, 1.1, 1, *p* < 0.05), homologous recombination (0.5, 0.4, 0.4, *p* < 0.01), nitrogen metabolism (0.5, 0.5, 0.4, *p* < 0.05), lipoic acid metabolism (*p* < 0.05), mismatch repair (*p* < 0.01), DNA replication (*p* < 0.01), and nicotinate and nicotinamide metabolism (*p* < 0.05).

## 4. Discussion

### 4.1. Sweet Potato Is an Optional Intercrop for Weed Control in Citrus Orchards

Compared to other cultivated mandarins (*C. reticulata* Blanco), the “Chachiensis” is a very special citrus variety. Its fruit flesh is usually dumped, but its pericarp have been used as a traditional medicine to treat chronic indigestion and respiratory diseases for hundreds of years [34,35]. Due to its antioxidant, lipid-lowering, and antimicrobial activities, its pericarp is also widely used as an important ingredient in daily cooking, drinking, and seasoning [34]. From these aspects, the usage of pesticides, including herbicides, is strictly controlled in the orchards of “Chachiensis”. In this study, the growth of sweet potatoes was very effective in the control of common weeds, which performed better than the well-known intercrop of soybeans. This might be because sweet potato is a herbaceous vine, its dense vines and leaves covered the orchard row space, thus restricting the growth of weeds [36,37]. In addition, sweet potato is abiotic stress-tolerant, which might have competitive advantages to the growth of weeds [36,37].

### 4.2. Intercrops Are Effective in Maintenance of Soil Nutrition

Firstly, intercropping significantly improved soil fertility, primarily by elevating soil organic matter—excluding the GS87. From previous studies, the root exudates of intercrops would add fresh organic matter directly to their rhizosphere soils [38,39] and increase the availability of soil organic carbon indirectly by changing the diversity and functions of soil fungal and bacterial communities [40,41]. The soils of GS87 did not accumulate more organic matter than those of the weeds (Table 1); this might due to the active bacterial respiration and other activities [42]. Accordingly, the soils of GS87 had a higher diversity of bacterial species and bacterial functional genes than those of the weeds (Figure 2B,D and Figure 7A,B). Secondly, the soil P, K (except PS32), and the beneficial metal elements were also commonly higher in the soils of intercrops than those of weeds. This was also commonly observed in other studies, reflecting that the intercropping system was more efficient in either the utilization or the conservation of soil nutrients than the natural growth of weeds [1,2]. The accumulation of P, K, and other beneficial nutrients in soil is primarily attributed to inputs from foliar fertilizers, root exudates, and plant litter, which are then mineralized by soil functional microbes, including phosphate-solubilizing bacteria [39,40,41]. Although root exudates of intercrops and weeds were not measured in this study, the fungal and bacterial community composition and structure differed among plant groups (Figure 3), suggesting that these groups may release distinct chemical compounds via root exudates. Subsequently, the fungi and bacteria enriched in response to root exudates from the intercrops mediate the decomposition of recalcitrant compounds, and the resulting nutrient release likely feeds back positively to soil nutrient accumulation [39,40,41].

However, the soil N only increased 0.1% to 0.2%, on average, in the soils of intercrops than those of weeds. Similarly, soil N was primarily supplied through plant root exudates and litter, both of which depend on soil microbes for transformation and deposition. In turn, the growth of plants and microbial activity also consume soil N, and this combined input–output dynamic may help maintain soil N content at a relatively stable level [12,13]. Even in the soils of leguminous intercrops, the increase of soil N was not significant. Accordingly, the relative abundances of N-fixing bacteria, like *Bradyrhizobium*, in the soils of soybeans were not dominant and not significant higher than the soils of weeds. A possible explanation for low N accumulation is that the soybean plants were not densely planted, and the soil lacked sufficient N-fixing bacteria. As a result, weeds thrived alongside the soybean plants and counteracted the N-accumulation benefits derived from their root systems.

### 4.3. Taxonomic and Functional Restructuring of Soil Microbiomes by Intercropping

Compared to the weeds, the intercrops significantly affected the composition and structure, and the major genera, rather than the richness and diversity, of the soil fungal and bacterial communities. The composition and structure of soil fungal and bacterial communities are mainly determined by the exudates of plant roots, which implies that different plant species shape their own rhizosphere microbial communities by their distinctive root exudates [39,40]. Compared to sweet potatoes, the intercrops of two soybean varieties shared more similar fungal and bacterial communities to the weeds. As we mentioned above, this might also reflect that the growth of the soybeans was contaminated by common weeds.

The fungal genera *Talaromyces* and *Penicillium* were positively affected by the intercrops. They are both from the class Eurotiomycetes, which includes abundant environmental saprophytes and opportunistic pathogens [43,44]. Therefore, their occurrence might be associated with the increased soil organic matter by the intercrops. For the bacterial community, the genera *Sphingomonas* and *Nocardioides* were found with both increased relative abundance and functional gene content by the intercrops, which implies their dominance and key functions in the soils of the intercropping systems. The bacteria of *Sphingomonas* are well-known for their multifaceted functions in soils, such as the degradation of organometallic compounds and the improvement of plant tolerance to abiotic stresses [45,46]. Interestingly, the bacteria of *Nocardioides* are “specialists” on the degradation of hard-to-degrade compounds, such as aromatic compounds, hydrocarbons, and haloalkanes [47]. In turn, the fungal genera *Trichosporon*, *Saitozyma*, and *Trichoderma*, and the bacterial genera *Chujaibacter*, *Acidothermus*, *Acidibacter*, *Conexibacter*, and *Mycobacterium*, were negatively affected by the intercrops. These microbial species might be out-competed by the enriched species when they were growing in the rhizosphere of the intercrops [7,12]. This suggests that shifts in fungal and bacterial communities, by the intercrops, are associated with soil nutrient accumulation, a process that is probably mediated by microbial decomposition of recalcitrant compounds from root exudates.

## 5. Conclusions

The citrus intercropping systems, with leguminous plants of soybean or with non-leguminous plants of sweet potato, are effective practices in control of common orchard weeds and accumulation of soil nutrients, such as P, Mg, Mn, and Zn. In comparison to weeds, the intercrops are deterministic in shaping soil fungal and bacterial composition, structure, dominant genera, and functional activities. The intercropping systems with both soybean and sweet potato are sustainable management practices in citrus orchards. The increased accumulation of soil organic matter, P, K, and other micronutrients may be achieved by the variation of fungal and bacterial communities, such as *Sphingomonas* and *Nocardioides*, as their relative abundances are improved in taxonomy and function by the intercrops. Further studies should focus on (1) characterizing the plant-growth-promoting functions of the enriched fungi and bacteria, and (2) evaluating their potential for field application in sustainable agriculture.

## Figures and Tables

**Table 1:** Soil physicochemical properties and metal contents of the sampled soils grown with the intercrops and weeds.

Soil Properties	Guangshu87 (GS87)	Pushu32 (PS32)	Huning95-1 (HN95-1)	Hongqiu1 (HQ1)	Weeds (Control)
pH value	4.63 ± 0.04	4.76 ± 0.05	4.20 ± 0.03	4.90 ± 0.04	4.69 ± 0.21
Organic matter (%)	3.44 ± 0.05	3.96 ± 0.08 *	3.77 ± 0.02 *	3.67 ± 0.03 *	3.52 ± 0.04
N (%)	0.25	0.24	0.24	0.24	0.23
P (mg/kg)	206.86 ± 12.67 *	147.61 ± 6.56 *	115.32 ± 5.38 *	129.29 ± 5.32 *	85.29 ± 3.03
S (mg/kg)	168.07 ± 22.85	149.89 ± 32.03	142.59 ± 8.71	127.58 ± 29.62	159.33 ± 34.73
K (mg/kg)	509.68 ± 38.85 *	281.30 ± 11.05	423.20 ± 37.96 *	399.53 ± 15.46 *	314.63 ± 19.34
Na (mg/kg)	11.59 ± 0.93	11.95 ± 0.45	11.88 ± 0.21	13.08 ± 0.38	11.44 ± 0.35
Ca (mg/kg)	767.75 ± 18.09 *	844.50 ± 4.96 *	522.19 ± 12.21 *	923.25 ± 4.42 *	576.17 ± 11.25
Mg (mg/kg)	198.13 ± 5.77 *	177.79 ± 1.07 *	152.27 ± 3.66 *	228.25 ± 1.38 *	146.54 ± 0.76
Fe (mg/kg)	263.50 ± 5.66 *	231.62 ± 2.59	247.55 ± 7.40 *	234.16 ± 3.77	229.54 ± 3.58
Mn (mg/kg)	56.16 ± 2.11 *	42.37 ± 0.59 *	38.58 ± 1.43 *	42.20 ± 0.58 *	33.36 ± 0.40
Zn (mg/kg)	4.08 ± 0.32 *	5.27 ± 0.22 *	4.41 ± 0.35 *	6.63 ± 0.69 *	3.85 ± 0.23
Cu (mg/kg)	2.92 ± 0.17	3.07 ± 0.16	2.92 ± 0.18	3.17 ± 0.06	2.88 ± 0.07

“*” indicates *p* < 0.05 compared to the weeds.

## Data Availability

Raw sequencing data are available from the first author (C.Z.) upon reasonable request.
